# Connections between expiratory bulbospinal neurons and expiratory motoneurons in thoracic and upper lumbar segments of the spinal cord

**DOI:** 10.1152/jn.01008.2012

**Published:** 2013-01-16

**Authors:** J. D. Road, T. W. Ford, P. A. Kirkwood

**Affiliations:** ^1^Sobell Department of Motor Neuroscience and Movement Disorders, UCL Institute of Neurology, London, United Kingdom;; ^2^The Lung Centre, Respiratory Division, Department of Medicine, University of British Columbia, Vancouver, British Columbia, Canada

**Keywords:** nucleus retroambiguus, abdominal motoneurons, bulbospinal connections, respiratory pathways

## Abstract

Cross-correlation of neural discharges was used to investigate the connections between expiratory bulbospinal neurons (EBSNs) in the caudal medulla and expiratory motoneurons innervating thoracic and abdominal muscles in anesthetized cats. Peaks were seen in the cross-correlation histograms for around half of the EBSN-nerve pairs for the following: at T8, the nerve branches innervating internal intercostal muscle and external abdominal oblique muscle and a more distal branch of the internal intercostal nerve; and at L1, a nerve branch innervating internal abdominal oblique muscle and a more distal branch of the ventral ramus. Fewer peaks were seen for the L1 nerve innervating external abdominal oblique, but a paucity of presumed α-motoneuron discharges could explain the rarity of the peaks in this instance. Taking into account individual EBSN conduction times to T8 and to L1, as well as peripheral conduction times, nearly all of the peaks were interpreted as representing monosynaptic connections. Individual EBSNs showed connections at both T8 and L1, but without any discernible pattern. The overall strength of the monosynaptic connection from EBSNs at L1 was found to be very similar to that at T8, which was previously argued to be substantial and responsible for the temporal patterns of expiratory motoneuron discharges. However, we argue that other inputs are required to create the stereotyped spatial patterns of discharges in the thoracic and abdominal musculature.

in the neurophysiology of respiration, expiration is often regarded as the poor relation of inspiration, even by authors who champion its “rediscovery” in the central pattern generator of the rat brain stem (Feldman et al. 2013). Like many others, these authors relegated expiration in eupnea to a passive mode, compared with the active mode for inspiration. In more demanding conditions (exercise, hypercapnia), all authors agree that expiratory muscles become active. However, even in eupnea in anesthetized animals, there is evidence from many studies for activity in at least some expiratory muscles (for references see de Almeida et al. 2010; also Leevers and Road 1995). Moreover, if one considers antecedent neurons in the pathway to expiratory motoneurons, the expiratory bulbospinal neurons (EBSNs), which are the neurons that convey the respiratory drive from the pattern generator to the spinal cord, it is clear that a strong control signal for expiration is transmitted to the spinal cord at all levels of respiratory drive. Even at a minimal level of respiratory drive, at the threshold for hypocapnic apnea, many EBSNs are active. Above this, their activity increases steeply with the CO_2_ level and more EBSNs are recruited (Bainton and Kirkwood 1979). The same neurons (the EBSNs) are also believed to transmit the commands for vital airway defensive behaviors, cough and the expiration reflex (for references see Baekey et al. 2004), as well as being involved in the commands for vomiting (Miller et al. 1987) and other motor acts.

This study investigates the nature of the linkages between EBSNs and both intercostal and abdominal motoneurons, which are presently not well documented. In the clinical domain, information about these pathways is relevant not only with respect to disorders of respiration, per se, but also for disorders of coughing and, most importantly, for efforts to restore cough when this ability is impaired, as in high spinal cord injury (Di Marco et al. 2009).

EBSNs in the cat comprise a major proportion of the neurons in the caudal nucleus retroambiguus (NRA) (Boers et al. 2005; Merrill 1970). Anatomic experiments have shown that the neurons of this nucleus in the cat not only project widely to spinal motor nuclei (Holstege 1991) but also make extensive direct connections to motoneurons at cervical, thoracic, and lumbar levels (Billig et al. 2001; Boers et al. 2006; VanderHorst at al. 1997). With physiological methods at thoracic segments, the EBSNs have been demonstrated to make strong monosynaptic connections to motoneurons, by cross-correlation (Cohen et al. 1985; Kirkwood 1995) and by intracellular spike-triggered averaging (Kirkwood and Sears 1973; Saywell et al. 2007). Connections were demonstrated to both intercostal and abdominal motoneurons (Saywell et al. 2007).

However, the connections to the abdominal motoneurons of the upper lumbar cord remain uncertain. In the only systematic study, Miller et al. (1985), using spike-triggered averaging of a peripheral nerve recording, observed only very sparse connections to motoneurons in L1, although similar effects were also shown for two EBSNs by Baekey et al. (2004). Moreover, with regard to EBSNs the anatomic evidence is only indirect, as emphasized by observations in the lower lumbar and sacral cord. For these segments, Boers et al. (2005) showed that the projections from the NRA included not only EBSNs but also many non-EBSNs, the non-EBSNs being more likely to project to the motor nuclei than the EBSNs.

The experiments described in this article clarify the situation by showing the presence of similar direct connections from EBSNs to thoracic and to upper lumbar expiratory motoneurons of various muscles, including connections to the two segmental levels from the same individual EBSNs. Preliminary reports have appeared (Kirkwood and Road 1995; Road and Kirkwood 1993), and some of the results were included in a review by Iscoe (1998).

## METHODS

### Preparations

Experiments were conducted according to United Kingdom (UK) legislation [Animals (Scientific Procedures) Act 1986] under Project and Personal Licences issued by the UK Home Office. The data are from 10 cats of either sex, weighing 2.1–3.85 kg, which were anesthetized with sodium pentobarbitone (initial dose 37.5 mg/kg ip, then iv as required), maintained under neuromuscular blockade with gallamine triethiodide (subsequent to surgery) and artificially ventilated via a tracheal cannula with oxygen-enriched air, to bring the end-tidal CO_2_ fraction initially to about 4%. CO_2_ was then added to the gas mixture to raise the end-tidal level to a value sufficient to give a brisk respiratory discharge in the midthoracic intercostal nerves (typically 6–7%).

We aimed to use a (surgically adequate) level of anesthesia in the range from light to moderately deep, as described by Kirkwood et al. (1982). Before neuromuscular blockade, a weak withdrawal reflex was elicited by noxious pinch applied to the forepaw, but not to the hind paw. When present, pinch-evoked changes in blood pressure (measured via a femoral arterial cannula) were only small and of short duration. During neuromuscular blockade, anesthesia was assessed by continuous observations of the patterns of the respiratory discharges and blood pressure together with responses, if any, of both of these to a noxious pinch of the forepaw. Only minimal, transient responses (similar to those before neuromuscular blockade) were allowed before supplements (5 mg/kg) of pentobarbitone were administered. The responses to a noxious pinch always provided the formal criteria, but in practice the respiratory pattern, indicated by an external intercostal nerve discharge that was continuously monitored on a loudspeaker from the induction of neuromuscular blockade, always gave a premonitory indication. Any increase from the usual slow respiratory rate (e.g., Fig. 5), typical of barbiturate anesthesia, led to such a test being carried out. The animal was supported, prone, by vertebral clamps at about T5 and T11, a clamp on the iliac crest, and a plate screwed to the skull. The head was somewhat ventroflexed. Rectal temperature was maintained between 37° and 38°C by a thermostatically controlled heating blanket. The bladder was emptied by manual compression at intervals. Systolic blood pressures were above 80 mmHg throughout. At the end of the experiment the animals were killed with an overdose of anesthetic.

**Fig. 1.**
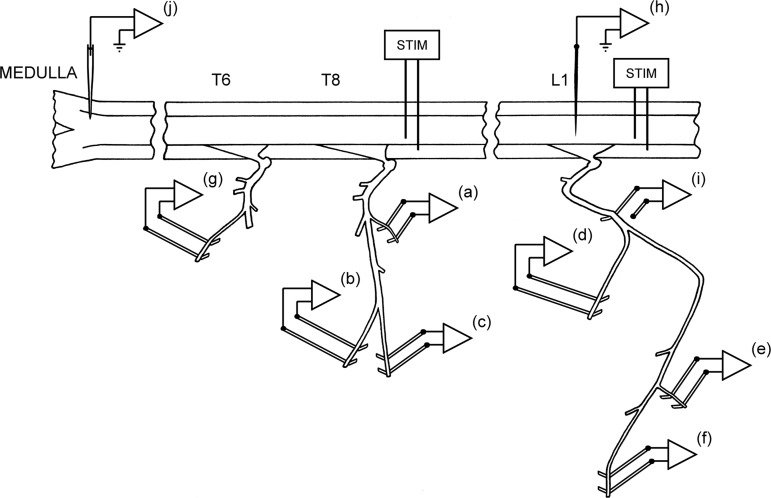
Experimental arrangement. Efferent discharges were recorded from 7 nerves or nerve branches: *a*, first filament of the internal intercostal nerve of T8 (T8Int); *b*, lateral branch of the internal intercostal nerve of T8 (T8EO); *c*, distal remainder of T8 internal intercostal nerve; *d*, nerve to external abdominal oblique at L1 (L1EO); *e*, one of the nerve branches to internal abdominal oblique at L1 (L1IO); *f*, a distal branch of the L1 ventral ramus, which included innervation of transversus abdominis (L1Dist); and *g*, external intercostal nerve at T6. The electrode (*i*) was used to record spike-triggered averaged axon potentials of motor axons to estimate peripheral conduction times in the L1 nerves, the tungsten microelectrode (*h*) was used to record averaged axonal potentials, terminal potentials, or focal synaptic potentials in L1 segment from individual expiratory bulbospinal neurons (EBSNs), and the glass microelectrode (*j*) was used to record spike trains from the EBSNs in the caudal medulla. Two pairs of stimulating electrodes (STIM) were used for antidromic identification of EBSN axons at different segmental levels.

Eight of the animals were used for cross-correlation measurements (the core measurements of the study), and the other two were control animals, used for estimations of projection frequencies and of variation in the conduction velocities of the bulbospinal axons in different segments. For the cross-correlation measurements, the following nerves were prepared on the left side for the recording of efferent discharges via platinum wire electrodes: at T8, one of the filaments of the internal intercostal nerve, which are the naturally occurring branches that leave the nerve at intervals to innervate the internal intercostal muscle layer (Sears 1964a), usually the most proximal one (Fig. 1*a*; T8Int), as in Bainton and Kirkwood (1979); the lateral branch of the internal intercostal nerve, which innervates external abdominal oblique (Fig. 1*b*; T8EO); and the distal remainder of the internal intercostal nerve (Fig. 1*c*; T8Dist), which innervates the more distal part of the internal and parasternal intercostal muscles, transversus abdominis, and rectus abdominis (for references see Meehan et al. 2004). Branches of the L1 ventral ramus were similarly prepared. A major branch separates from the nerve proximally (underneath the large axial muscles, longissimus dorsi and iliocostalis) and can be found emerging at the lateral border of iliocostalis to innervate external abdominal oblique from the external surface, together with a cutaneous branch. Either the major branch itself, or the portion innervating the muscle was prepared (Fig. 1*d*; L1EO). The remainder of the ventral ramus runs on the external surface of transversus abdominis and was exposed for 20–30 mm by dissecting away the overlying two layers of abdominal oblique muscles. Within that distance, two or three branches were usually seen to innervate the immediately overlying internal abdominal oblique; one of these was prepared (Fig. 1*e*; L1IO). Innervation of internal oblique muscle was confirmed by the observation of a local twitch on cutting the branch. Of course it is possible that the branch went on to innervate another layer (perhaps projecting back again to transversus abdominis), but this seems unlikely. In addition, one of the more distal branches (usually the most lateral in our dissection) was also prepared (Fig. 1*f*; L1Dist). Usually transversus abdominis was seen to twitch when this was cut, but it is possible that the internal oblique layer also did so. Both of these layers are likely to have been innervated by this branch (Fregosi et al. 1992), and rectus abdominis may also have been included. Finally, the external intercostal nerve of T5 or T6 (Fig. 1*g*) was also prepared, whose efferent discharges were used to define the timing of central inspiration.

Laminectomies of about one segment length were made to expose a thoracic and a lumbar segment of the spinal cord, usually T9 and L1–L3. Stimulating electrodes (a pair of cut-back glass-insulated tungsten microelectrodes) were inserted into the left spinal cord at each of the two levels. The laminectomy and nerves at the thoracic level were submerged in a single paraffin oil pool constructed from skin flaps. The lumbar nerves were recorded under petroleum jelly, most often with a piece of thin plastic film separating the electrodes from underlying muscle. In three animals, a patch of pia was removed from the dorsum of L1 segment, and a glass-insulated, platinum-black coated tungsten microelectrode (A. Ainsworth, Northampton, UK) (Merrill and Ainsworth 1972), mounted on a micromanipulator, was inserted into the spinal cord (Fig. 1*h*) to record terminal or focal synaptic potentials from selected EBSNs, using extracellular spike-triggered averaging (extracellular STA) (Munson and Sypert 1979). Recording sites for this were chosen to be in or near the ventral horn, as indicated by antidromic field potentials from stimulation of the peripheral nerves (Kirkwood 1995). In the same three animals, STA was also used to assess the efferent conduction times in the peripheral nerves. For this, a proximal part of the L1 abdominal nerve was exposed (just caudal to the transverse spinal process of L2 vertebra), and a platinum wire recording electrode (Fig. 1*i*) was placed on or under the nerve incontinuity (reference on nearby muscle). The signal from this was averaged, using triggers from the efferent discharges. Similar measurements were made for the recordings from the two distal nerve branches at T8 in two animals. In the two control animals, the thoracic laminectomy was extended to include T11, and a pair of stimulating electrodes were inserted into this segment, in one experiment instead of the lumbar pair, and in the other in addition to it. In all animals, an occipital craniotomy was made, the dura opened, and a small patch of pia removed from the right side of the medulla.

### Recording Procedures

A glass microelectrode filled with 3 M NaCl (broken back to a tip diameter of 3.0–3.2 μm) was introduced into the medulla through a hole in a small pressure plate to record, via a conventional amplifier, the extracellular activity of single units in the right caudal medulla (Fig. 1*j*). Units were located around 2.5 mm caudal to obex and 1.8–2.8 mm from the dorsal surface of the medulla, within or very close to the NRA, as indicated by the prominent narrow column of multiunit expiratory activity (Merrill 1970). EBSNs with axons descending on the left side were identified by antidromic responses to stimuli (0.1-ms pulses) delivered via the T9 spinal cord stimulating electrodes. Identification was confirmed by a collision test (at 2× threshold) and by double stimuli to show that the minimum interval in the collision test was not due to soma refractoriness. Each axon was then also tested for antidromic activation at the other spinal cord stimulation site(s).

Activity from the single unit and the efferent discharges of all 7 nerves were recorded over periods of 25–100 min on magnetic tape. Subsequently, the times of occurrence of spikes in the medulla and nerves were acquired for cross-correlation analysis and analyzed for connections between the bulbospinal neuron and motoneurons as described by Davies et al. (1985a), using the criteria set out by Kirkwood (1995) to reject the smaller amplitude spikes, likely derived from gamma motoneurons. The EBSN and efferent discharge recordings were bandpass filtered, usually at 300 Hz to 3 kHz, the ventral horn recordings used for STA were filtered at 10 Hz to 10 kHz, and the proximal whole nerve recordings were filtered at 300 Hz to 10 kHz.

### Analysis

The single-unit nature of the EBSN recording was always confirmed by the absence of very short intervals in an autocorrelation plot (for example, see Fig. 5*B*, *top left*). The value of the interval from the first peak or the shoulder of the auto correlation histogram was used to calculate an approximate modal firing frequency of the EBSN (Ford et al. 2000; Kirkwood 1995). Delays in the collision test, latencies in the extracellular STA (see Fig. 3*A*), and lags in the cross-correlation histograms (see Figs. 5 and 6) were all referred to the early rising phase of the (main) negative-going phase of the trigger spikes.

Orthodromic conduction times for the EBSNs were calculated from the collision test as the critical delay minus 0.5 ms (Davies et al. 1985a; Kirkwood 1995), and conduction velocities were calculated from these values, together with distances from the medulla to the stimulating electrodes or between pairs of stimulating electrodes. Distances were measured postmortem by exposing the whole relevant length of the spinal cord and laying a thread along it, as were distances from the spinal cord to the nerve recording electrodes. The EBSN conduction time to T8, the “axonal time” (Davies et al. 1985a; Kirkwood 1995), was calculated as the fraction of the time to the T9 stimulating electrodes in proportion to the relative distance to rostral T8. For estimations of the conduction times to L1, see results.

Cross-correlation histograms were constructed over ±24 ms with a bin width of 0.192 ms. Peaks in the histograms were accepted as significant if a single bin exceeded 3.29$\sqrt{m}$ (Sears and Stagg 1976), where *m* was the baseline count, as described by Davies et al. (1985a) and Kirkwood (1995). Single bin counts exceeding this limit (which will sometimes occur by chance) were ignored if they fell outside the range of expected latencies (0–10 ms; Davies et al. 1985a). In cases of a sloping baseline, which could occur on account of the variable respiratory phasing of the discharges (for instance, a positive slope could occur if the motoneuron firing generally phase-lagged the EBSN discharge), a sloping straight line was fitted by eye and *m* was taken as the value on that line at the time of a peak (Davies et al. 1985a, 1985b; Kirkwood 1995; Vaughan and Kirkwood 1997). The strength of a peak was assessed by *k*, the ratio of the highest count within a peak to *m* (Davies et al. 1985a, 1985b; Kirkwood 1995; Sears and Stagg 1976). The sensitivity of detecting a peak of a given value of *k* depends on *m*, and therefore on the length of the recording and the discharge rates of the EBSN and of the efferent motoneuron population, all of which were highly variable between the different nerves and between different runs. To give some meaning to an absence of any peak, only histograms with *m* ≥ 121 were considered. A baseline count of 121 will allow the detection of peaks with *k* ≥ 1.3 (cf. Davies et al. 1985a, 1985b; Kirkwood 1995).

Mean values are presented with ±SD. In statistical tests, *P* < 0.05 was taken as significant.

## RESULTS

### EBSN Properties and Calculations of Axonal Conduction Times

EBSNs were recorded in two types of experiments, first in two control animals where only projection frequencies were investigated (*n* = 34), and second in those where the connections from EBSNs to motoneurons were assessed (*n* = 27). The conduction velocities from the medulla to T9 were similar for the two groups: ranges were 22–113 m/s (mean 66.5 ± 18 m/s) and 42–94 m/s (mean 68.1 ± 14 m/s), respectively. These values were also similar to those previously reported from this laboratory (Ford et al. 2000; Kirkwood 1995; Saywell et al. 2007) and by Sasaki et al. (1994).

**Fig. 2.**
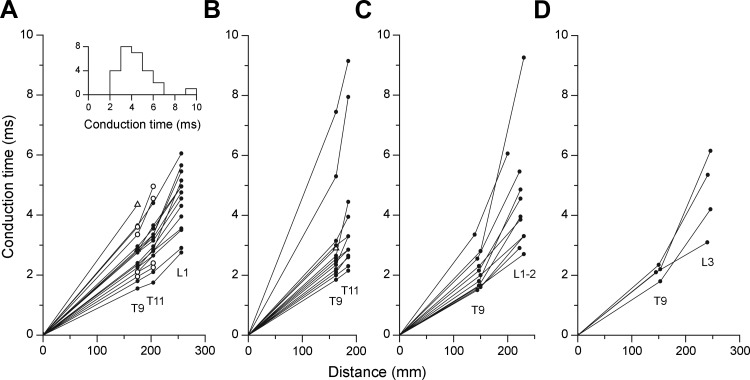
Axonal conduction times for EBSNs. Each graph shows the conduction times from individual EBSNs, calculated from the collision tests in the indicated segments and plotted against the distance from the medulla. *A* and *B*: measurements in the 2 control animals, used only for EBSN projection frequency and conduction time measurements (one animal in *A*, the other in *B*). Filled circles, antidromic identification at all sites tested; open triangles, units not activated from T11; and open circles in *A*, units not activated from L1. *Inset* in *A* shows a histogram of conduction times to L1 or rostral L2 (includes data from *A* and *C*). *C* and *D*: measurements from animals used for cross-correlation measurements. Only units antidromically identified from L1–L3 are included (“L1–2” refers to L1 or rostral L2). Data are from 3 animals in each of *C* and *D*.

In the first of the two control animals, 21 EBSNs were identified from stimulation at T9. Nineteen of these were found to also project to T11, and 15 of these went on to project to L1. Figure 2*A* shows the conduction times of the EBSNs to the different segments. These measurements are important in helping to estimate the axonal conduction times to L1 for the connectivity measurements (see below). It can be seen in Fig. 2*A* that most of the axons showed marked slowing of conduction between T11 and L1, but only a few showed marked slowing between T9 and T11. Note also that two of four of those not identified at L1 (i.e., terminating between T11 and L1) were among those showing marked slowing between T9 and T11. The experiment in the second control animal was designed to test simply how many of those EBSNs identified from T9 also projected to T11, originally as a control for a different set of experiments (Ford et al. 2000). These data are of interest because they confirm the high proportion of axons that do project to T11 (12/13 in this animal, a very similar proportion to that in the first animal). Furthermore, most of these (Fig. 2*B*) showed relatively little slowing of conduction up to T11, again very similar to those illustrated in Fig. 2*A*.

In the main series of 8 animals, 27 EBSNs were identified from T9. Ten of these were tested and positively identified from caudal L1 or rostral L2 (3 animals), and 9 were tested from L3 (3 animals), with 4 of these being identified. The remaining eight EBSNs (4 animals) were not tested by stimulation at a lumbar site, although two of them were identified as projecting to L1 by the presence of terminal potentials (TPs) and focal synaptic potentials (FSPs) in STA recordings.

The conduction times for the 14 axons identified by stimulation at lumbar sites are plotted in Fig. 2, *C* and *D*. A slowing of conduction between T9 and the lumbar sites, compared with medulla to T9, is evident for most axons, similar to that in Fig. 2*A*. The slowing may be related to termination of some axons in the upper lumbar cord (note that a lower proportion of axons were found to project to L3 than to L1 or L2; cf. Miller et al. 1985), and much of this slowing could therefore have occurred within the lumbar cord, consistent with the relatively minor slowing between T9 and T11. Thus estimations of the conduction times to the L1 motoneurons (likely to be mostly in rostral L1; Meehan et al. 2004) are rather uncertain from these data alone, especially when the stimulating electrodes were at L3.

**Fig. 3.**
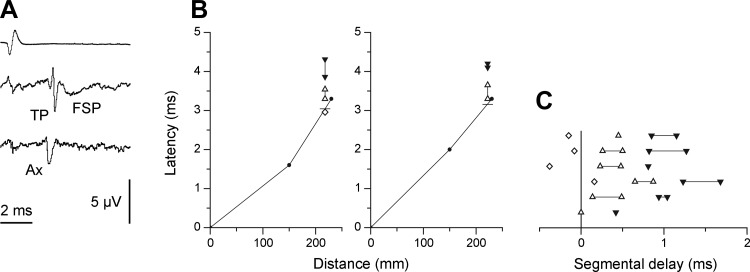
Use of extracellular spike-triggered averaging to confirm EBSN axonal conduction times to L1 and to estimate segmental delay in L1. *A*: examples of an axonal potential (Ax), a terminal potential (TP), and a focal synaptic potential (FSP) recorded in the ventral horn at L1, averaged with respect to the spikes of an EBSN (trigger spike shown in *top* trace). The lowest trace (Ax) was averaged at a depth of 3.2 mm, and the middle trace (TP and FSP) at a depth of 2.6 mm, 4,096 sweeps for each. The voltage calibration applies to the two lowest traces. *B*: latencies of averaged waveforms from 2 EBSNs compared with their axonal times, plotted as in Fig. 2. The unit illustrated at *left* gave an axonal potential (latency shown by open diamond); the unit at *right* did not. Open triangles joined by vertical lines indicate the latency range for TPs at different recording sites; filled triangles indicate equivalent ranges for FSPs. The distance coordinate for these points indicates the actual distance from the medulla to the sites used for the averaged recordings. Short horizontal lines show the interpolated value assumed for the axonal time to these sites. *C*: measurements similar to those in *B*, summarized for all 6 EBSNs where comparisons were made with conduction times from the collision tests. The abscissa (segmental delay) indicates the observed latencies with respect to the interpolated axonal time, with each row representing 1 EBSN. Symbols are as described in *B*.

STA recordings were made in L1 to clarify the situation. Recording sites yielding TPs and FSPs were found for eight axons, and for four of these, at least one site giving an axonal potential was also found (for criteria see Munson and Sypert 1979; Schmid et al. 1993). Figure 3*A* shows a typical example, with an axonal potential evident at a relatively deep recording site, probably in or near the white matter, and with a later TP and FSP occurring more dorsally. Figure 3*B* shows the timings of these components for two other EBSNs, one where an axonal potential was identified and one where one was not. The latencies of the STA events were formally related to the data from the axonal stimulation by defining the axonal time here by a linear interpolation between the conduction times for the two stimulation sites plotted against distance, using the distance value corresponding to the STA recording site (short horizontal lines indicate the interpolated axonal times in Fig. 3*B*).

The equivalent measurements for all six EBSNs for which both collision data from a lumbar site and STA measurements were available are summarized in Fig. 3*C*, which shows latencies with respect to the axonal times (segmental delays). The first point to note is that the segmental delays of three of four of the axonal potentials were negative, the mean value for the four potentials being −0.11 ms. Moreover, for the two EBSNs without axonal potentials, the TPs also had relatively short segmental delays. These generally short latencies presumably occur as a result of progressive slowing in EBSN conduction velocities with distance, consistent with the relatively modest slowing between T9 and T11, thus leading to an overestimation of conduction time by the use of linear interpolation. Despite this, the segmental delays for most of the TPs and FSPs were slightly longer than equivalent measurements in thoracic segments. For comparison, we have considered means of the midpoints of the segmental delay ranges for the TPs and for the FSPs for each EBSN, shown in Fig. 3*C* (midpoints were used instead of means for individual EBSNs because the sites surveyed with STA were not systematic between different EBSNs). These gave values of 0.38 and 1.03 ms, respectively, which can be compared with mean values in comparable measurements from Ford et al. (2000), which were 0.34 and 0.82 ms, respectively (control population, rostral and caudal sites combined).

### Peripheral Conduction Times

**Fig. 4.**
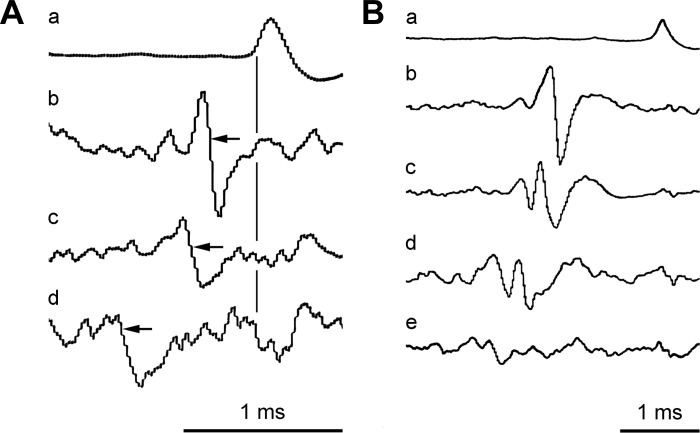
Spike-triggered averaging for estimating peripheral conduction times. Trigger spikes (*a*) were efferent spikes recorded at the cut end of the nerve; the other traces show averages from the nerve at a more proximal site. *A*: T8EO nerve. Selected trigger spikes: *b*, large alpha; *c*, small alpha; *d*, gamma. Gains are arbitrary, except gain for *d* (2,048 sweeps) was 2 times gain for *b* and *c* (each 1,024 sweeps). *B*: L1Dist nerve. Selected trigger spikes: *b*, large alpha; *c*, medium-sized alpha; *d*, small alpha; *e*, gamma (spike amplitude range overlapped with that for *d*). Gains are arbitrary, except gain for *d* (1,024 sweeps) was 2 times gain for *b* and *c* (each 512 sweeps); gain for *e* (2,048 sweeps) was 4 times gain for *b* and *c*. Conduction time measurements are illustrated in *A* for each of the groups of efferents detected (times from vertical line to points of arrows).

For the thoracic segments in Kirkwood (1995), the conduction times in the motoneuron axons formed only a minor part of the “transmission delay” (the difference between the axonal time and the start of a correlation histogram peak). For these segments, the conduction distance from the cord to the recording electrodes was around 15–20 mm, representing a conduction time of around 0.2 ms. The recordings from the internal intercostal nerve filaments in the present study were made at a similar distance, but for the other two nerves at T8, the distances were longer. The effects of this were estimated by measuring the conduction times directly by STA. Figure 4*A* shows averaged recordings made from the internal intercostal nerve of T8 (at a distance of 16 mm from the spinal cord) triggered by the efferent spikes recorded from the T8EO, 20 mm more lateral. Either larger or smaller groups of (assumed) alpha motoneuron spikes were selected, along with the smallest group, assumed to be spikes of gamma motoneurons. The latencies of the averaged waveforms (−0.28, −0.40, and −0.86 ms, as indicated) thus gave conduction velocities of 70, 50, and 23 m/s. Corresponding values for T8Dist in this animal were 84, 58, and 50 m/s. Similar values were measured in one other animal. Thus one might expect transmission delays from these more distal recording sites to be longer by around 0.2 or 0.3 ms than at the proximal sites. Also, the larger temporal dispersion in the different motoneuron axons would be likely to increase the duration of the (multiunit) histogram peaks (see *Cross-Correlation Results*) by around 0.1 ms.

Larger differences were involved at L1, with the conduction distances from the spinal cord ranging between 69 and 93 mm for the recording sites on L1IO and L1Dist in the three animals where STA was used. Figure 4*B* shows the averaged recordings from the proximal site on the L1 ventral ramus triggered from spikes in L1Dist, involving three different amplitude ranges of assumed alpha spikes and one range of assumed gamma spikes. Note that, as in Fig. 4*A*, spikes of individual units were not selected as triggers, but nevertheless action potentials from individual axons (or groups of axons) can be distinguished in the averages. Overall, four different conduction velocities could be derived: 54, 50, and 41 m/s for three groups taken as alpha motoneurons and 34 m/s for the gamma motoneurons. For L1IO, the values of conduction velocities were 36, 33, and 27 m/s for presumed alpha motoneurons and 18 m/s for the gamma motoneurons. Similar ranges were observed for these two nerves in two other animals, but in the one animal where there were sufficient presumed alpha motoneuron discharges in the L1EO nerve, the values were 81 and 73 m/s for the alphas and 22 m/s for the gammas. The conduction velocities for L1IO and L1Dist appear rather slow for alpha motoneurons, but there could be two factors to explain this. First, the former nerves were recorded at a quasi-intramuscular site, so axonal branching within the nerve trunk (Eccles and Sherrington 1930) would have been very likely, and second, these nerves had been exposed for a large fraction of their conduction distance and may well have been below core temperature. In contrast, the recording site for the L1EO nerve was proximal to its entry into EO muscle, and this nerve ran below the thick axial muscles up until this point. Whatever the cause, the calculated conduction times from the spinal cord for the presumed alpha motoneurons were relatively long, with values for L1IO of 1.42–1.98, 1.73–2.18, and 2.51–3.33 ms for the three animals investigated. For L1Dist the ranges were 1.12–1.57, 1.15–1.4, and 1.70–2.24 ms. Note, therefore, not only that the conduction times were considerably, and variably, lengthened compared with the recordings at T8 but also that temporal dispersion within an individual nerve recording could have been considerable (0.37 to 0.82 ms in these examples).

### Cross-Correlation Results

**Fig. 5.**
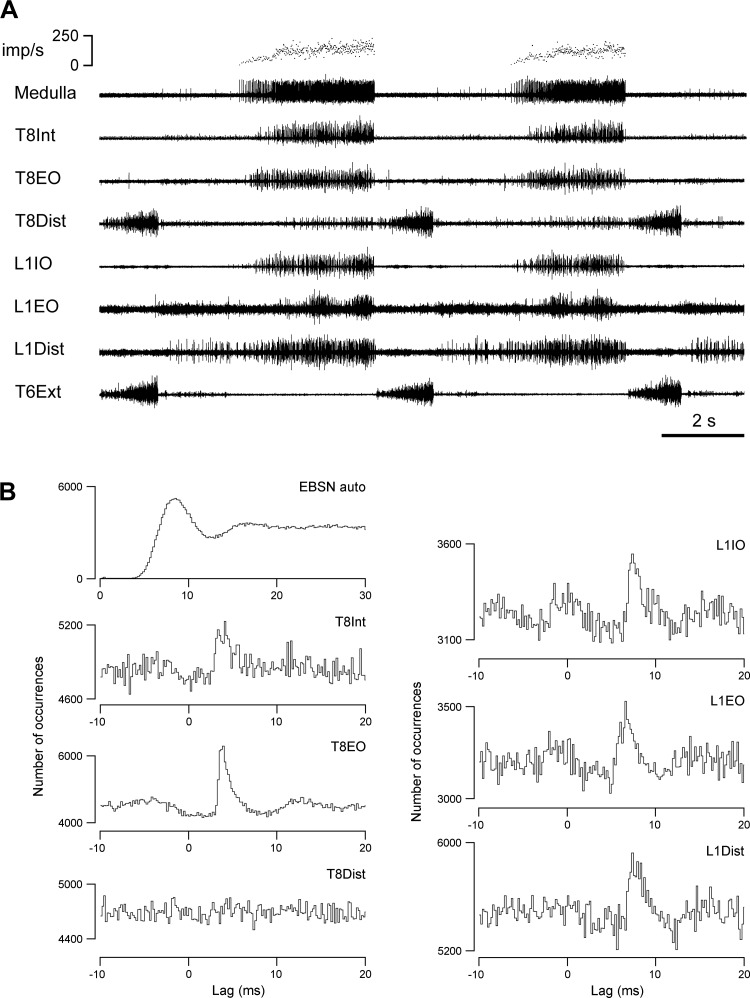
Example of recordings used for cross-correlations together with the cross-correlation histograms calculated from the recordings. *A*: extract from recordings of the efferent discharges in all 7 nerves, plus an EBSN (medulla). *Top* trace indicates firing rate of the EBSN, shown as instantaneous frequency. *B*: *top left* histogram shows autocorrelation of the EBSN spike train; the other 6 histograms show cross-correlations between the presumed alpha motoneuron discharges (response spikes) from the indicated nerves and the EBSN (reference spikes). Length of run, 4,039 s (172,922 EBSN spikes).

An example of the efferent discharges recorded from all six nerves used for cross-correlation measurements, together with the recordings from the external intercostal nerve and an EBSN, is shown in Fig. 5*A*. The recordings are typical in that all six nerves show bursts of large-amplitude spikes (presumed alpha motoneurons) in expiration, with T8Dist also including an inspiratory burst. This latter presumably represents the innervation of the parasternal interchondral muscle, which has an inspiratory activation pattern and an inspiratory action (De Troyer et al. 2005; Taylor 1960). The discharges within expiration for all expiratory channels also usually showed periodic variation, with a period of 1.15 s (52 min^−1^). This represents a stretch reflex from the ventilator. The intensity of the expiratory bursts varied between preparations, including variation in the relative levels of activity in the different nerves. For instance, in Fig. 5*A*, the intensity of the expiratory discharge in T8Dist is relatively low compared with usual, but that in the L1EO nerve is relatively high. This latter nerve often showed only small-amplitude tonically firing spikes, which were taken as gamma motoneurons and were not selected for the cross-correlations. The small spikes are also visible as the irregular baseline in the L1EO recording in Fig. 5*A*. The overall variation in activity is reflected in the number of EBSN-nerve combinations that were counted as tested. Only those with *m* ≥ 127 were included in this group (see methods). The presence or absence of peaks in the correlation histograms are therefore described below separately for the different nerves.

#### T8 histograms.

**Fig. 6.**
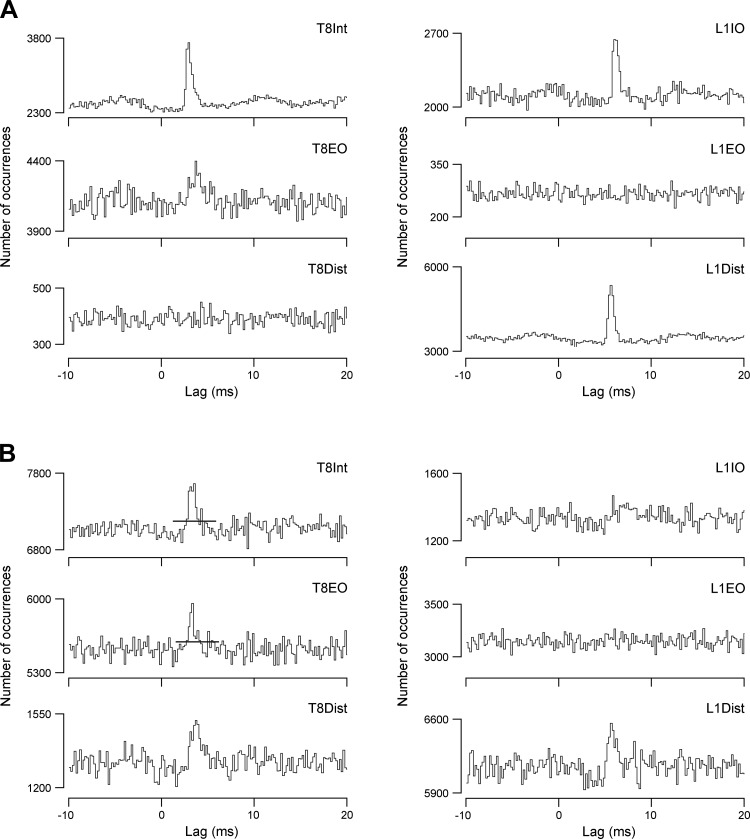
More examples of cross-correlations. Histograms are calculated from 2 different EBSNs and the 6 expiratory nerves, as indicated, for one EBSN in *A* and the other in *B*. The horizontal lines in T8Int and T8EO histograms in *B* indicate the raised level of *m* adopted for these 2 histograms, to take account of a presumed minor medium-width component (note the small early “foot” on these peaks). Lengths of runs: *A*, ∼4,750 s (64,755 EBSN spikes); *B*, 4,000 s (213,475 EBSN spikes).

**Fig. 7.**
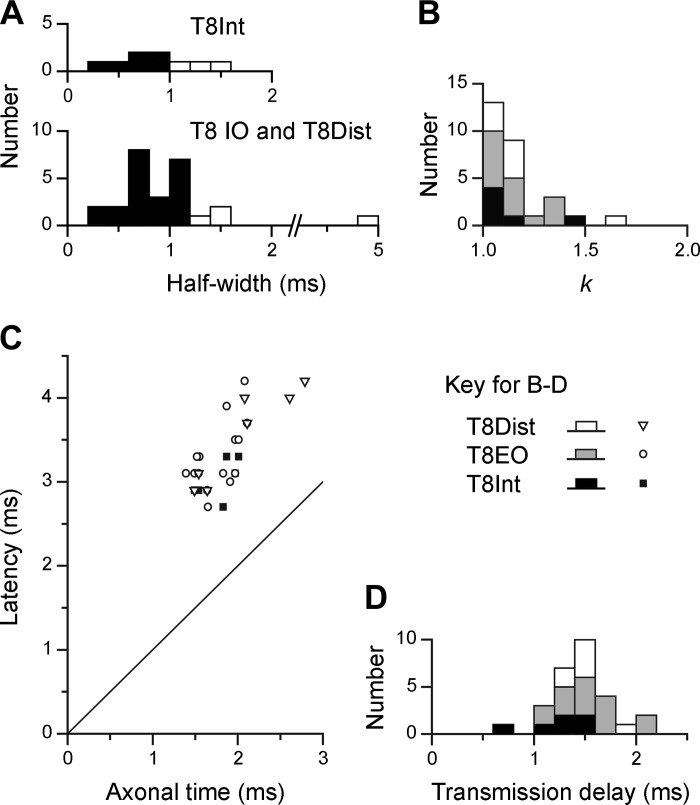
Characteristics of cross-correlation histogram peaks for T8 nerves. *A*: half-widths of all significant peaks. Filled bars, narrow peaks; open bars, medium-width peaks. *B*: distribution of amplitudes of narrow peaks (*k* = maximum value/baseline value). *C*: latencies of narrow peaks plotted against axonal time for the EBSN. The line of equality is also plotted. *D*: transmission delays (differences between latencies and axonal times) for narrow peaks. Symbols and shading for the 3 nerves are indicated. Half-widths and transmission delays were measured to 0.1 ms but are displayed in 0.2-ms bins in *A* and *D*.

Twenty-seven EBSNs were tested for connections to one or more of the T8 nerves, and significant cross-correlation peaks were seen for at least one of the nerves for 18 of these EBSNs. Examples are included in Figs. 5 and 6. To assess the connections that these might represent, the latencies and durations of the peaks were measured and criteria similar to those of Kirkwood (1995) applied. The duration was assessed by the half-widths of the peaks (their durations at half-amplitude), the purpose mainly being to discriminate against presynaptic synchronization, likely to arise from synchrony between the EBSN investigated and other EBSNs that may synapse on the motoneurons (Davies et al. 1985a; Kirkwood 1995). As in those studies, for the T8Int nerves, we adopted a dividing line of 1.1 ms for the widest half-width acceptable for a peak to be ascribed to monosynaptic excitation (for justification, see Kirkwood 1995). For the other two nerves, the criterion was relaxed to 1.2 ms, on account of the expected additional temporal dispersion in these nerves (see above). Peaks narrower than these limits are referred to as “narrow peaks” and wider ones as “medium-width” peaks. Clear examples of narrow peaks from the thoracic nerves include T8EO in Fig. 5*B* and T8Int in Fig. 6*A*. One example of a medium-width peak is T8Int in Fig. 5*B*, assessed as having a half-width of 1.3 ms. Further features consistent with presynaptic synchronization for this peak are *1*) the small “foot” preceding the main part of the peak and *2*) the asymmetry of the periodic secondary peak, which is related to the periodic firing of the EBSN (see autocorrelation histogram in Fig. 5*B*) and which was only obvious to the left of the main peak. The absence of a similar peak to the right is good evidence for presynaptic synchronization (Moore et al. 1970). In contrast, the narrow peak from T8EO from the same EBSN (half-width of 1.2 ms) had symmetrical secondary peaks and was accepted as evidence of monosynaptic excitation. The histogram from the third thoracic nerve tested with this EBSN (T8Dist) showed no peak. For the two EBSNs represented in Fig. 6, the significant peaks were all classified as narrow, which was the most common type, overall. However, note that for two of these (T8Int and T8EO in Fig. 6*B*), the peaks were interpreted as being a narrow peak superimposed on a weak underlying medium-width peak. Values of half-width and *k*, together with the assessment of significance, were therefore all measured with respect to a slightly elevated baseline, representing a point on the assumed medium-width peak, as indicated. Distributions of half-widths of the correlation peaks from the thoracic nerves are included in Fig. 7*A* (filled columns: narrow peaks).

The proportions of EBSN/nerve pairs where the histograms showed significant peaks are listed in the first line of Table 1 and the proportions of narrow peaks in the second line, which represents the same numbers but after the histograms with medium-width peaks have been removed. There were no medium-width peaks for T8EO, but there were four for T8Int and three for T8Dist. These cannot be counted as representing the absence of a narrow peak, because it is not possible to say that a medium-width peak does not include a narrow component. Thus, as in Kirkwood (1995), these examples were counted as “not tested.” The end result represents the proportion of EBSN/nerve pairs deduced to show a monosynaptic connection. This is higher for T8EO (15/24) than for the other two nerves (6/17 and 7/19), but not significantly so (χ^2^, *P* > 0.05). The mean values of *k* for the narrow peaks are then listed in the third line of Table 1. The mean is higher for T8EO than for the other two nerves, but, as is clear from the distributions of *k* (Fig. 7*B*), not significantly so.

**Table 1.** Proportions of EBSN/nerve pairs giving significant histogram peaks, with amplitudes

	Thoracic			Lumbar		
	T8Int	T8EO	T8Dist	L1IO	L1EO	L1Dist
All EBSNs						
Significant peaks	10/21	15/24	10/22	10/24	3/15	10/27
Narrow peaks (or equivalent)	6/17	15/24	7/19	10/24	2/14	10/27
*k* (for narrow peaks)						
Mean (SD)	1.15 (0.16)	1.20 (0.16)	1.13 (0.05)	1.14 (0.06)	1.17 (0.10)	1.15 (0.14)
EBSNs with identified L1 projection						
Narrow peaks (or equivalent)	5/10	13/16	4/11	9/14	1/7	7/16

Values are proportions of EBSN/nerve pairs giving significant histogram peaks, together with the amplitudes of those peaks, assessed by *k*, for all expiratory bulbospinal neurons (EBSNs; *n* = 27) and for EBSNs with identified L1 projection (*n* = 16). T8Int, first filament of T8 internal intercostal nerve; T8EO, lateral branch of T8 internal intercostal; T8Dist, distal remainder of T8 internal intercostal; L1IO, one of the nerve branches to L1 internal abdominal oblique; L1EO, nerve to L1 external abdominal oblique; L1Dist, distal branch of L1 ventral ramus, which included innervation of tranversus abdominis. See text for further explanation.

Confirmation that the narrow peaks represented monosynaptic connections was obtained from their latencies, which are shown in Fig. 7*C*, plotted against the axonal time (the calculated arrival time of the EBSN impulse at rostral T8). There is an obvious relationship between the two, with the latencies being at an approximately constant distance above the line of identity, i.e., the difference between the latencies and axonal times being approximately constant. Distributions for this difference the “transmission delay” (Davies et al. 1985a) are shown in Fig. 7*D*, with the following means: T8Int, 1.25 ± 0.20 ms (*n* = 6); T8EO, 1.54 ± 0.31 ms (*n* = 15); and TDist, 1.51 ± 0.20 ms (*n* = 7). The value for T8Int is very close to that from Kirkwood (1995) (mean 1.24 ms), whereas the differences between the values for T8Int and the other two nerves (0.27 and 0.26 ms) correspond closely to the range for the efferent conduction time difference described above (0.2–0.3 ms).

#### L1 histograms.

**Fig. 8.**
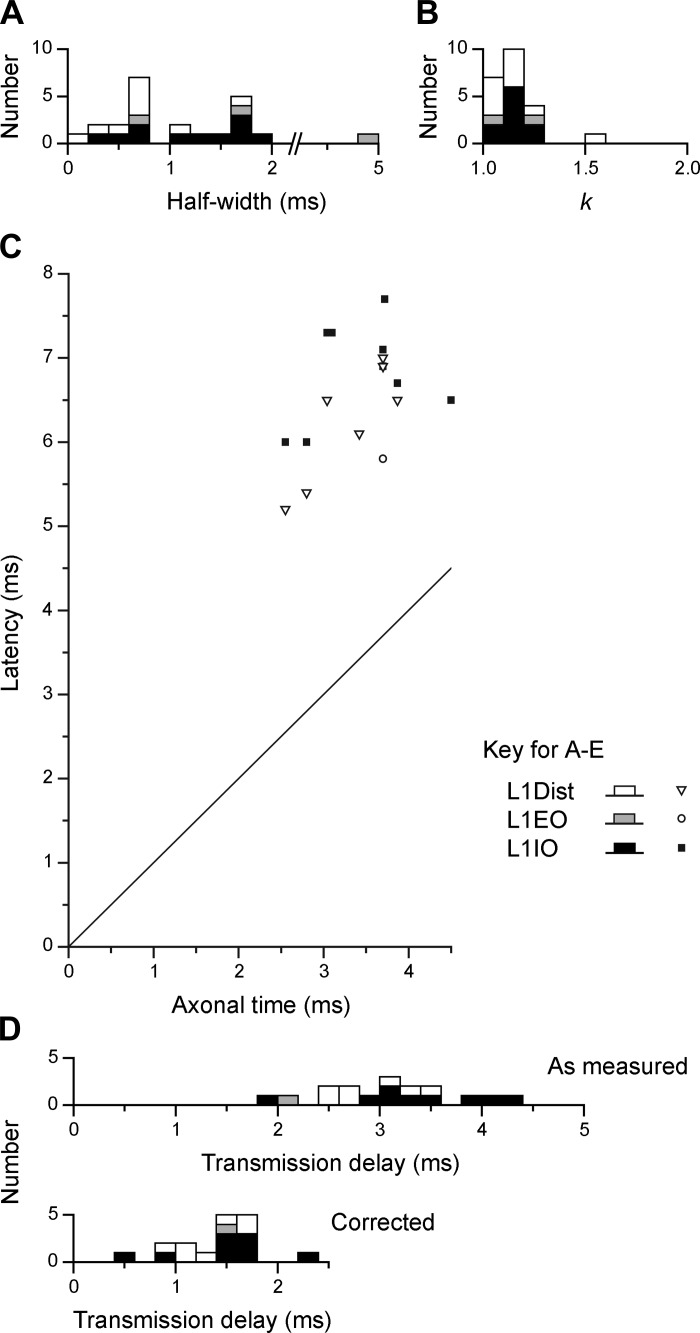
Characteristics of cross-correlation histogram peaks for L1 nerves. *A–D*: data are plotted as described in Fig. 7, except that the only peak taken as medium-width was the one with a half-width of 5 ms; all the others were accepted as equivalent to narrow peaks (see text). An additional distribution is included in *D*, *bottom* histogram, which shows “corrected transmission delays,” where the estimated peripheral conduction time for each nerve has been subtracted (see text).

**Fig. 9.**
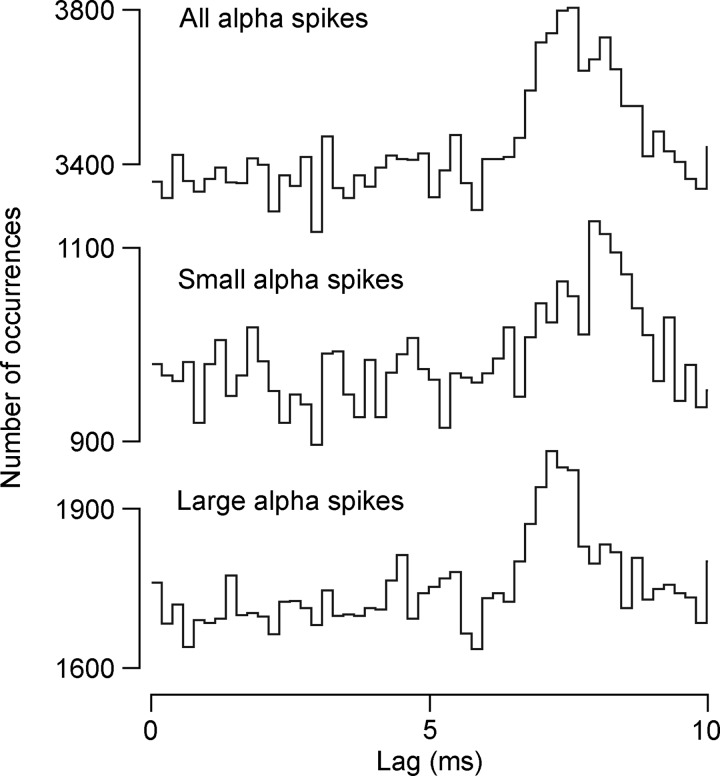
Detection of different components in a cross-correlation histogram peak for an L1IO nerve, according to efferent spike size. Spike sizes were selected via a window discriminator as indicated. Ranges of amplitude for small and large alpha spikes were nonoverlapping, with a gap between. Histograms are shown on an expanded time scale compared with Figs. 5 and 6.

The same 27 EBSNs were also tested for connections to one or more of the L1 nerves, and significant cross-correlation peaks were seen for at least one of the nerves for 13 of these EBSNs. All but one of these also showed peaks for one or more of the T8 nerves. Examples are included in Figs. 5, 6, and 9. As for the thoracic nerves, many of these peaks were narrow (e.g., 3/4 of those in Fig. 6), but all three of those in Fig. 5 had half-widths >1.1 ms, as was true for 10/23 of the total (for distribution, see Fig. 8*A*). However, we have classified only one of these as medium-width. This peak, although significant (1 bin), was relatively weak and noisy, with a half-width of about 5 ms. The other eight were not classified as medium-width, for the following reasons. First, the criterion of having a half-width >1.1 ms was chosen by Kirkwood (1995) because 1.1 ms represented the half-width of the distribution of conduction times of EBSNs to a thoracic segment (also see Davies et al. 1985a). For L1, that distribution must be wider than for T8 (Fig. 2, *A* and *C*), although the numbers of axons in the present study are rather small for an accurate estimation of a half-width as done previously. However, an estimate of around 2–3 ms is reasonable (see *inset* in Fig. 2*A*). Furthermore, peripheral temporal dispersion would be expected to make the minimum half-width for a medium-width peak at L1 even wider. In any case, apart from the one exception already mentioned, all of the observed histogram peaks for the L1 nerves were narrower than this, the widest half-width being 1.9 ms.

Second, the expected peripheral conduction times give an alternative explanation for the wider peaks. This was investigated by constructing cross-correlation histograms separately for small and large alpha motoneuron spikes within a given nerve recording. Such a procedure can only be carried out if the connections are sufficiently strong to still give clear peaks after the baseline counts have been reduced by the spike selection and was thus performed for the four peaks that showed the highest signal-to-noise ratio. We defined this as the height of the peak above the baseline, divided by the SD of the baseline counts, where the SD is given by $\sqrt{m}$, on the assumption of a Poisson distribution of counts in the baseline (Kirkwood and Sears 1982), i.e., (*k* − 1)*m*/$\sqrt{m}$ = (*k* − 1)$\sqrt{m}$. This took values of 4.5–7.5 for these four peaks. One of these is shown in the *top* histogram of Fig. 9, with the lower two histograms being derived from the two subgroups of spikes. In place of a peak with a half-width of 1.6 ms from the whole population, the two subgroups gave half-widths of 1.2 and 1.0 ms, and the main parts of their peaks differed in latency by 1.0 ms. A similar result was seen for the other three EBSN/nerve pairs for which a similar analysis was performed; subgroups could be defined, where in each case the smaller amplitude spikes gave peaks with longer latencies than for the larger amplitude spikes. The latency differences for these three other examples were 0.4, 0.8, and 0.8 ms (original half-widths were 1.2, 1.7, and 1.8 ms, respectively, subgroup half-widths 0.9 and 0.8 ms, 1.3 and 1.2 ms, and 0.6 and 1.3 ms, respectively).

All but one of the histogram peaks for the L1 nerves are therefore consistent with an origin in a monosynaptic connection and are thus included in the second line of Table 1. Note, however, that these totals include 11 EBSNs without confirmation of a projection to L1. Thus, for comparing connectivity with T8, the proportion of histograms with peaks for L1 should exclude these 11 EBSNs, as shown in the final line of Table 1. The proportions for L1IO and L1Dist (9/14 and 7/16, respectively) may then be seen to be similar to those for the T8 nerves, but that for L1EO is lower (1/7). It should be remembered, however, that for this nerve the expiratory-phased discharges were thin, at their best, and the efferent spikes contributing to the histograms may well have included a reasonable proportion of tonic, nonrespiratory discharges. The proportions for the excluded 11 EBSNs were lower, 1/10, 1/7, and 3/11 for L1IO, L1EO, and L1Dist, respectively, but this should be expected, of course, because not all of these EBSNs would have projected to L1. These peaks were nevertheless included in the population described for duration (above) and for *k*. Values of *k* were very similar to those at T8 (Table 1; also see the distribution in Fig. 8*B*).

Confirmation of monosynaptic connections was also sought from the latency measurements at L1, in the same way as for T8. Figure 8*C* is a similar plot to Fig. 7*C*, except that here the axonal time was calculated by linear interpolation from the measured values at T9 and L1–L3, as described with regard to Fig. 4. The general result is similar to that at T8, except that the transmission delays at L1 were longer and more variable (Fig. 8*D*, *top*). One inevitable contribution to these delays is the peripheral conduction time. The majority of the peaks (13) came from recordings in the three animals in which STA had been used to measure peripheral conduction velocities. For these 13 peaks, the calculated conduction times from the spinal cord root entry for the fastest fibers observed by STA (1.12–2.51 ms) were subtracted from the measured transmission delays to give “corrected transmission delays.” Another two peaks came from recordings in a fourth animal, where the distances were known and a conduction velocity of 55 m/s was assumed, giving a conduction time (for both nerves) of 1.4 ms. For the remaining two peaks, where neither distance nor conduction velocity were known, a conduction time of 1.5 ms was assumed. These values were then subtracted to give the corrected transmission delays for those remaining four peaks, and the values for all 17 peaks are shown in Fig. 8*D*, *bottom*. The values lie within the same range as for the T8 values (Fig. 7*D*), but the mean (1.44 ± 0.39 ms) is 0.2 ms larger than the mean for T8Int. Note, however, that the value for T8Int was not corrected for conduction delay from the spinal cord (a conduction time of about 0.2 ms). The real difference in corrected transmission delay between L1 and T8 is therefore 0.4 ms. The issue will be considered further in the discussion, but for the present, this difference is considered not sufficient to doubt the conclusion that the histogram peaks for the L1 nerves, just like those at T8, do indeed represent a monosynaptic connection from the EBSNs to L1 motoneurons.

The only remaining issue is whether there is any pattern discernible in the connections, particularly for T8 compared with L1. For this we should consider only the 16 units identified as projecting to L1 (final line of Table 1). All of the EBSNs in this group that gave connections to L1 also showed connections at T8. However, 4/16 of the EBSNs with connections at T8 showed none at L1. Two EBSNs showed no connections at either level, although one of these was tested with a total of only two nerves and the other with only 3 nerves, and both involved low counts in their histograms.

**Fig. 10.**
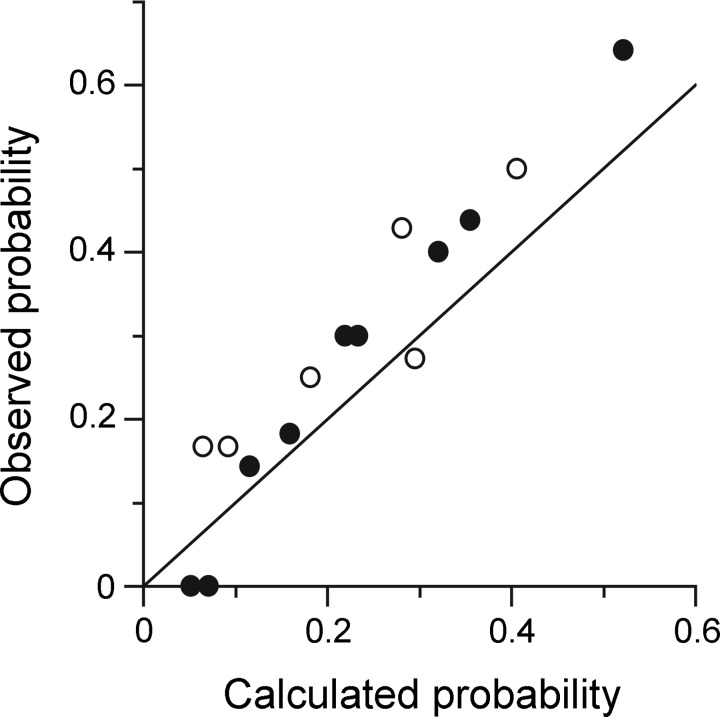
Apparent lack of pattern in the observed connections. Graph shows the frequency of observing connections for an EBSN to both of a given pair of nerves (e.g., T8Int and L1Dist) when both of the nerves were tested (“observed probability”), plotted against “calculated probability,” which is the product of the frequencies of finding a connection from an EBSN to each of the 2 nerves separately (i.e., the probability expected by random association). For 6 nerves, there are 15 pairs, represented by the 15 points (open circles, nerves from the same segment; filled circles, one nerve in T8 and one in L1). The line of identity is also plotted. See text for more explanation.

As apparent from Figs. 5 and 6, various combinations of connections were seen, as summarized in Table 2. Table 2 shows first the numbers of nerves tested (at least 4 nerves for 13/16 of the EBSNs) and second the number of connections seen (fairly evenly spread across 0 to 5 nerves), the most common (4 instances) being connections for three nerves. Within this distribution, no common pattern was obvious. It seemed as if the combinations of nerves in pairs, where each pair showed a connection, occurred at random. This possibility was checked by comparing the probabilities of pairs of nerves both showing a connection from an EBSN, with the probabilities expected by random association. For instance, there were 4 EBSNs showing connections to both T8Int and L1IO out of 10 EBSNs tested for both nerves, a probability of 0.4, compared with the multiplied probabilities from Table 1: 5/10 × 9/14 = 0.321. A similar comparison was made for each of the 15 possible pairings available from 6 nerves, and the results are plotted in Fig. 10. No pairing stands out as being grossly different from the random prediction, although nearly all of the points lie above the line of identity, i.e., there is a general small increase in probability of connections being seen for both nerves of a given pair above that predicted by chance.

**Table 2.** Numbers of EBSNs tested with indicated numbers of nerves, and numbers of EBSNs giving narrow peaks (or equivalent) for indicated numbers of nerves

No. of nerves tested		1	2	3	4	5	6
No. of EBSNs		0	1	2	4	5	4
No. of nerves giving peaks	0	1	2	3	4	5	6
No. of EBSNs	2	3	3	4	2	2	0

## DISCUSSION

This study has demonstrated, for the first time, substantial direct (monosynaptic) excitation of abdominal motoneurons in the upper lumbar spinal cord from EBSNs in the caudal medulla, connections that in almost all respects are similar to the well-established connections to the motoneurons of the thoracic cord. The result is of no surprise; previous studies have demonstrated projections of EBSNs to the ventral horn of these segments, both by anatomic (Billig et al, 2001; Boers et al. 2006; Holstege 1989; Miller et al. 1989) and by physiological means (Miller et al. 1985; Sasaki et al. 1994), but, for reasons set out in the Introduction, the connections could not be assumed to be present without direct measurements, nor was there any estimate of the possible strength of such connections. This study provides both verification of the connections and an estimate of their strength. We also confirm, for firing motoneurons, the result previously obtained via intracellular STA by Saywell et al. (2007), that the monosynaptic connections at thoracic levels include motoneurons of all three nerve branches T8Int, T8EO, and T8Dist.

The only previous study that systematically investigated the possibility of direct connections for the upper lumbar segments (Miller et al. 1985) found very infrequent connections (seen in only 4% of trials). The explanation for the failure of these authors to find the connections that we have seen most probably lies in the methods they used (STA from the whole abdominal nerve discharge). The amplitudes of the two responses that they did find (their Fig. 7), together with their quoted amplitude for baseline activity, can be used to calculate *k* for these responses, giving values of 1.25 and 1.5. These values are at the upper end of the range of values we measured (see Fig. 8*B*). Thus, if the majority of connections in their preparations were similar to those in ours, they may well have given signals too small to detect against noise (the larger of their 2 responses had a signal-to-noise ratio of only around 2). The likelihood of this could be even greater, since even though these authors did ensure, by the use of an expiratory load, that an expiratory discharge was present in the nerve, their nerve recordings must have included gamma motoneuron discharges, inevitably diluting any response from alpha motoneurons. Note that the experiments of Miller et al. (1985) employed unanesthetized, decerebrate preparations. Thus the explanation put forward by Saywell et al. (2007) for a previous discrepancy between their positive evidence for EBSN connections vs. largely negative evidence from Merrill and Lipski (1987), that of a different level of anesthesia, should not apply here.

### Nature of the Connections

Our conclusion depends on the interpretation of peaks in the cross-correlation histograms for EBSNs and lumbar nerve discharges as representing monosynaptic connections. We are confident that this interpretation is correct, but it should be emphasized that the evidence is not as absolute as it is for the thoracic segments (Davies et al. 1985a; Kirkwood 1995, Saywell et al. 2007). In those earlier studies, which were also confirmed by the measurements for T8 segment in the present study, the two important features of the cross-correlation peaks, their durations and their timings [and their equivalents for excitatory postsynaptic potentials (EPSPs)], both fitted well the expectations for monosynaptic connections, even though discrimination had to be made against medium-width peaks or synchrony potentials, which arise from presynaptic synchrony. The upper limit for the half-width of a histogram peak considered safe for not arising by presynaptic synchrony (1.1 ms) was coincidently about the same as that expected for the longest duration peak arising from a monosynaptic connection (Davies et al. 1955a; Kirkwood 1995). About half (12/22) of the significant cross-correlation peaks observed for L1 discharges in the present study had durations within that limit, so for these, no question of presynaptic synchrony arises. For the others, their durations were longer than would be expected from the rise times of known single-axon EPSPs in motoneurons. On the other hand, because of slowed conduction in the population of EBSNs that we assume to be the source of presynaptic synchrony (Davies et al. 1985a; Kirkwood 1995), the expected duration of correlation peaks from this source actually should be longer than the observed durations. Thus temporal dispersion in the peripheral axons, which was shown to be a sufficient explanation for the four instances investigated (Fig. 9), should be accepted as being the most likely explanation. The peaks in the correlation histograms in the subpopulations (Fig. 9, *B* and *C*) both showed a remaining broader base. This could represent either a small component of presynaptic synchrony or, more likely, imperfect separation of efferents with different conduction velocities.

Next, we should consider the latencies of the peaks. After appropriate correction for peripheral conduction times, the mean transmission delays were only 0.4 ms longer than the transmission delays at T8, which themselves were appropriate to monosynaptic excitation. None of the L1 delays were too short, which is also consistent with a lack of effect from presynaptic synchrony (Davies et al. 1985a; Kirkwood 1995). Thus the only other possibility that we suggest needs to be considered is a possible disynaptic connection. The longest single value of corrected transmission delay (2.25 ms) could allow for this, but, as has been consistently argued in publications from this laboratory, although one can almost always make particular assumptions to construct alternative explanations for any one individual cross-correlation peak, the important judgments are made with respect to the properties of the group. In the present study there were no indications of a separate group that could represent a disynaptic linkage. In contrast, in recent measurements of similar connections from EBSNs to thoracic motoneurons in the rat (de Almeida and Kirkwood 2006), the transmission delays fell into two nonoverlapping groups, a presumed monosynaptic group for internal intercostal nerve motoneurons, with a mean of around 1.1 ms, similar to that in the present study for T8Int, and a presumed disynaptic group for external intercostal nerve motoneurons, with a mean of around 2.6 ms.

The small increase in transmission delay that we did observe at L1 compared with T8 could have a variety of explanations, all within the context of the monosynaptic connection. First, although the sample was small and nonsystematic, the mean segmental delays for the FSPs at L1 were around 0.2 ms longer than previously reported for T8. Second, this itself could be an underestimate. The measurements of segmental delays in the present study were based on axonal times calculated for the actual distance along the cord to the FSP recording site. The transmission delays were calculated on the basis of axonal times to the rostral end of the segment. Because of the slowed conduction in the EBSNs at L1 compared with T8, the delays within the segment would be expected to be, on average, longer at L1 than at T8. Finally, the peripheral conduction time used to give the corrected transmission delay was that for the fastest observed efferent axons. The particular motoneurons involved in the cross-correlation peaks may have had somewhat slower axons.

These factors also all contribute to the variation in the delays observed, and, of course, this variation is one of the reasons why some individual peaks may never be unequivocally assigned to a monosynaptic connection. By the same token, as noted previously (Kirkwood 1995; Saywell et al. 2007), we can never totally exclude a minor contribution in our measurements from either disynaptic connections or from presynaptic synchrony. However, for all the reasons above, we believe these contributions will be minor and can safely be ignored so that our selected cross-correlation peaks can reasonably be assigned, as a group, to monosynaptic connections.

### Strength of the Connections

We are claiming that the overall connection is substantial. How does this compare with that at thoracic levels, where the intracellular data (Saywell et al. 2007) allowed estimations to be made of its functional significance? First, we can see that the levels of *k* were very similar, both in comparing T8 and L1 in this study and in comparing the values derived from the nerve branches at T8 in the present work and the whole nerves in Kirkwood (1995). Second, with the exception of L1 EO, the occurrence of a connection was also similar for the same comparisons. With regard to L1EO, as mentioned in results, the phasic, presumed alpha motoneuron discharges in this nerve were generally very weak, but the tonic, presumed gamma motoneuron discharges were strong, so it is quite likely that the selection of spikes for cross-correlation from the EO recordings inadvertently contained a higher proportion of gamma spikes than intended, and this low occurrence is likely to be an underestimate, or at best an unreliable sample.

One feature of the cross-correlation peaks was different in comparing T8 with L1, namely the increased durations at L1. This means that the values of *k* for the wider peaks also represent an underestimate. For instance, in Fig. 9, had there been less peripheral dispersion, the separate peaks for the two subgroups of motoneurons would have superimposed and would therefore have summed to give a value of *k* about twice that observed. Note that we have chosen not to measure the area under the peak as a measure of correlation strength, for the same reason as given in Kirkwood (1995), that an area measurement is much more affected by a small degree of presynaptic synchrony than an amplitude measurement. Overall, therefore, the strength of connections for the tested EBSNs, at least for L1IO and L1Dist, should be regarded as somewhat stronger than at T8.

To give a direct comparison of the total EBSN input to the sampled motoneurons at L1 compared with thoracic levels, we need to consider two other factors: *1*) the proportion of EBSNs projecting to L1 compared with T8, and *2*) the proportion of the connections from axons descending on the right (i.e., from EBSNs ipsilateral to the motoneurons), which was included in the assessments by Kirkwood (1995) and Saywell et al. (2007). For the first factor, 15/21 of the EBSNs in the present study that projected to T9 went on to caudal L1 (Fig. 2*A*) and 32/37 of the EBSNs in the study by Sasaki et al. (1991) projected as far as L2, giving a total of 47/58 (81%). For the ipsilateral projection of EBSNs, which takes place via collaterals at the spinal level (Kirkwood 1995), there is no direct physiological information for L1, but evidence of a bilateral projection from the NRA to the upper lumbar motor nuclei (segments L1–L3 in the different studies) has been common to all the anatomic tracing studies (Feldman et al. 1985; Holstege 1989; Holstege and Kuypers 1982; Miller et al. 1989; VanderHorst and Holstege 1995; VanderHorst et al. 1997). The projection may be even more strongly bilateral at L1 than at thoracic levels (see illustrations in Holstege 1989). Thus, considering all the factors, with the presence of fewer axons projecting to L1 being compensated by the temporal dispersion in some L1 cross-correlation histograms and perhaps also by an increased ipsilateral projection, we can conclude that the monosynaptic input to L1, at least to L1IO and L1Dist motoneurons, is as large as that to thoracic expiratory motoneurons.

### Functional Aspects

This result, that the direct connections from EBSNs to L1 expiratory motoneurons are substantial and similar in strength to those at thoracic levels, needs to be put in context. One possible comparison is with the direct connections from inspiratory bulbospinal neurons (IBSNs) to phrenic motoneurons, where the individual connections from individual IBSNs appeared similar in strength and occurrence to those in the present study, when measured using the same techniques (Davies et al. 1985b). Estimating the overall strength of connections to phrenic motoneurons depends, as in this study, on assumptions concerning the number of participating neurons. These may have been underestimated by Davies et al. (1985b), and this number is in any case debatable, according to which categories of the IBSNs are included (for references see De Troyer et al. 2005), but most authors would regard this connection, IBSNs to phrenic motoneurons, as being strong. Nevertheless, as argued by Saywell et al. (2007), the connections from EBSNs to thoracic motoneurons may, on their own, provide only around one-third of the depolarization required to bring the motoneurons into their firing range, at least in the anesthetized animal. We should assume this is also likely to be the case for the L1 motoneurons; yet, many of the abdominal motoneurons at L1 are firing with an expiratory pattern under these conditions (Fig. 5). Because the expiratory ramp components of the central respiratory drive potentials (CRDPs; Sears 1964b) in their study were also relatively modest (the largest was 6 mV in amplitude), Saywell et al. (2007) deduced that the additional input required was likely to come from tonic rather than phasic expiratory neurons. Although information on CRDPs is not available for the L1 motoneurons, we can argue by analogy and suggest that the same is likely to be true for them (but see below). In an unanesthetized animal, other mechanisms, such as voltage-dependent amplification of the CRDPs via persistent inward currents, will also come into play, but these, too, like interneuron circuits, may well be subject to local modulation (Enríquez Denton et al. 2012).

The expiratory discharges of thoracic motoneurons have a stereotyped spatial pattern across the thorax (De Troyer et al. 2005). Because there was no specificity apparent either in connections to individual motoneurons of different muscles or in CRDPs, Saywell et al. (2007) deduced that the spatial patterns of the activity were also likely to depend on tonic inputs. Do the present measurements further contribute to estimates of the specificity of the connections to different groups of expiratory motoneurons? Not really: a major problem, not applicable to the STA recordings of Saywell et al. (2007), is that the cross-correlation measurements can only be made for firing motoneurons, thus biasing the detection of connections to the nerves with the stronger discharges. It might be tempting to conclude that the commonly observed lower expiratory activation of EO muscle compared with that of the inner layers (also consistent with the generally low level of phasic L1EO nerve activity in the present study) is determined principally by a low connectivity of EBSNs to motoneurons of L1EO, compared with L1TA and L1Dist. However, for the above reason, we think this would be unwise. Moreover, as also mentioned above, the apparently lower connectivity to L1EO motoneurons may not be a reliable observation. Rather, we want to emphasize the apparent randomness in the shared projections here (Fig. 10), which is consistent with nonspecific connections. The small increase in probability of occurrence of connections above chance from one EBSN to any pair of nerves could be the result of any factor that influenced the number of baseline counts in both histograms and thus influenced the threshold for detecting a peak. These factors would have included the length of the recording, the firing frequency of the EBSN, and the general excitability of the preparation.

We therefore suggest, by analogy with measurements in the thoracic cord (Saywell et al. 2007), that the EBSN input supplies a precise temporal pattern of excitation as a background input to all expiratory motoneurons, thoracic and lumbar, but that other inputs are responsible for determining the degree to which any particular muscle or region of muscle becomes activated. A virtue of this conclusion is that it readily allows for the great variation in patterns of abdominal activity reported for different states such as anesthesia, chemical drive, and posture, etc. (Iizuka 2011; Iscoe 1998; Pagliardini et al. 2012). The pattern of discharge of the EBSNs may be relatively constant and does not need to vary for EBSNs projecting to different motor nuclei. A further virtue of this conclusion is that it allows a resolution of an apparent contradiction with regard to abdominal motoneuron activity in the retching movements of vomiting. As already pointed out (Kirkwood and Road 1995), the result here of a direct connection from most of the EBSNs to abdominal motoneurons (i.e., those supplying T8EO) seems inappropriate to retching, where the abdomen contracts with the diaphragm, yet the majority of EBSNs remain firing in the opposite phase (Miller et al. 1987). This is easier for the spinal cord circuits to deal with if the excitation from EBSNs is insufficient on its own to depolarize the motoneurons to threshold. Thus quite different mechanisms might operate to provide this depolarization in different behaviors. For vomiting, additional inputs could be recruited, such as the “non-EBSNs” in the NRA demonstrated by Boers et al. (2005), which are silent in the cat under barbiturate anesthesia. These neurons were shown to have projections to the ventral horn of lower lumbar segments but also could well participate in the anatomically defined projection to motoneurons in L1 (Billig et al. 2001; Boers et al. 2006). For cough, where all the expiratory motoneurons are likely to fire together, the EBSNs, when synchronized, might be sufficient to recruit the motoneurons to fire (but cf. Oku et al. 1994). However, to provide enough depolarization for the high firing rates involved, one could also suggest additional recruitment in the NRA. A candidate group here could be those neurons in the “non-EBSN” category that demonstrated subliminal respiratory modulation (Boers et al. 2005).

Returning to the question of recruitment during expiration, what other sources of inputs might be available? With regard to our preferred hypothesis of tonic inputs, or at least inputs that were nonrespiratory under the conditions of our experiments, inputs from muscle afferents (not necessarily monosynaptic) are obvious candidates (Leevers and Road 1995) (note the stretch reflex components obvious in Fig. 5*A*). Given the well-recognized postural role of abdominal muscle, any central inputs related to posture then could also be considered, such as those in the medial reticular formation, which may be activated from the vestibular system and which were shown anatomically to project to abdominal motoneurons (Billig et al. 2001). It is interesting that these authors suggested that these connections might “set the background excitability of respiratory motoneurons…, whereas respiratory group neurons elicit specific contractions of respiratory muscles…,” i.e., the opposite of our own hypothesis above. In fact, the justifications for both views are similar (apparently widespread “nonspecific” projections). A resolution of the problem awaits the identification of a set or sets of neurons with well-defined specific projections, selectively activated in different behaviors. One factor influencing any hypothesis is that many of the medial reticular formation neurons may be inhibitory (which those in the NRA are not). Another factor is that these reticular formation neurons are particularly well suited to be involved also in other phasic motor acts, such as vomiting or cough (for references see Billig et al. 2001).

With regard to our less favored, but still possible, hypothesis of additional phasic expiratory inputs, first, the many expiratory interneurons in the thoracic cord, particularly those with an additional tonic component to their discharges (Kirkwood et al. 1993), could be candidates. Many of these have long descending axons, but it is not known how many might project as far as L1. Second, much interest recently has been generated by the expiratory actions of neurons in the rostral medulla in the parafacial-retrotrapezoid region in the rat (Abdala et al. 2009; Pagliardini et al. 2011). Could they be contributing to some of the expiratory activity in our preparations, especially because we employed hypercapnia, as did Abdala et al. (2009)? There are a number of uncertainties here, especially in thinking of such activity as providing an additional input. First, although the retrotrapezoid nucleus is present in the cat and includes some expiratory neurons (Connelly et al. 1990), it is unknown whether it can operate in the cat as an independent expiratory oscillator, as in the rat. Second, even in the rat, this behavior is unlikely to be seen under barbiturate anesthesia (for references see de Almeida et al. 2010). Finally, as far as is known, this nucleus in the rat in any case sends its excitation via the NRA. Elements of this linkage were demonstrated directly in the neonatal rat by Janczewski et al. (2002), and EBSNs in the adult can also be deduced to receive the input from the rostral medulla by virtue of their strong, late expiratory “quantal” activation (de Almeida et al. 2010; de Almeida and Kirkwood 2010).

The patterns of abdominal nerve discharges were not themselves a focus of the present investigation. Nevertheless, some features are worth reporting. For comparison with other studies, note that the conditions of the present experiments consisted of a suspended, prone posture, with hypercapnia, intact vagi, neuromuscular blockade, and ventilation with a fast rate and low stroke volume. One feature that was observed in the present work that has not, to our knowledge, been reported before was that T8EO almost always showed a high level of phasic expiratory activity (note the high proportion of EBSNs counted as “tested” in Table 1). This might seem to contradict earlier studies that showed relatively low levels of expiratory electromyographic (EMG) activity in EO muscle, except that in most of these studies the positions of the EMG electrodes were typically placed relatively caudal on the abdomen (e.g., De Troyer and Ninane 1987; Kelsen et al. 1977). To our knowledge, accurate data on the regional innervation of EO muscle from individual segmental nerves have not been published, although S.-I. Sasaki (personal communication) has observed strictly segmented responses in the cat to stimulation of each individual nerve innervating the muscle (T6–L3). Thus the thoracic nerves innervate the more rostral regions and the lumbar nerves the more caudal regions. A few studies have included regionally selective abdominal recordings, and in particular some observations in humans, especially when upright, could correspond to our observations by demonstrating more respiratory activity rostrally in the abdomen and more tonic, posture-dependent activity caudally (De Troyer 1983; Strohl et al. 1981; but cf. Hoit et al. 1988). It should be added that the presence in these studies of instances of recordings showing tonic activity without any expiratory modulation means that the idea of a widespread nonspecific direct EBSN input to all abdominal motoneurons cannot be totally universal, at least in humans.

Our observations of strong tonic gamma motoneuron activity in L1EO are also consistent with the idea of a tonic, postural role for the caudal abdomen, even if this postural activity also has a respiratory function (De Troyer 1983; Grimby et al. 1976) The high level of tonic gamma motoneuron activity is also consistent with that reported by Russell et al. (1987) and could correspond to the high proportion of gamma motoneuron axons reported for the L1EO nerve by Niwa et al. (2008).

This study was introduced in terms of known connections in the cat. Are the results relevant to other species and other motor systems? First, similar patterns of activity to those in the cat are also found in the dog and in humans (De Troyer et al. 2005). Second, very similar patterns of anatomic projections have been reported from the NRA in a primate (VanderHorst et al. 2000), so the current measurements may well be directly relevant in primates in general, and thus in the human. Details, no doubt, will be different in different species (Iizuka 2011). One example is the tonic abdominal activity mentioned above. Another example comes from the rat, where similar monosynaptic connections to internal intercostal motoneurons have been found, even if rather weaker, but with the addition of a disynaptic connection to external intercostal motoneurons (de Almeida and Kirkwood 2006). These are consistent with the rather different patterns of activity in that species (de Almeida et al. 2010; de Almeida and Kirkwood 2010). However, the most general importance of the present measurements is that they extend what was shown for thoracic motoneurons by Saywell et al. (2007), i.e., that a set of connections that might be regarded by most investigators to be a strong direct command input from long descending fibers to a pool of motoneurons nevertheless still represents only a partial determinant of the pattern of activation within that pool. We suggest that this is a generalization likely to be true for all mammalian motor systems.

## GRANTS

This work was supported by the Canadian Medical Research Council, the Jeanne Anderson Fund (Institute of Neurology), and Wellcome Trust Grant 038027/Z/93/Z/1.5.

## DISCLOSURES

No conflicts of interest, financial or otherwise, are declared by the authors.

## AUTHOR CONTRIBUTIONS

J.D.R. and P.A.K. conception and design of research; J.D.R., T.W.F., and P.A.K. performed experiments; J.D.R. and P.A.K. analyzed data; J.D.R., T.W.F., and P.A.K. edited and revised manuscript; J.D.R., T.W.F., and P.A.K. approved final version of manuscript; P.A.K. drafted manuscript.
